# Abrasion Behavior of Different Charcoal Toothpastes on Human Dentin When Using Electric Toothbrushes

**DOI:** 10.3390/dj10030046

**Published:** 2022-03-11

**Authors:** Nadin Osmanaj, Svea Petersen, Michael Eisenburger, Andreas Greuling

**Affiliations:** 1 Department of Prosthetic Dentistry and Biomedical Materials Science, Hannover Medical School, Carl-Neuberg-Straße 1, 30625 Hannover, Germany; nadin.osmanaj@web.de (N.O.); eisenburger.michael@mh-hannover.de (M.E.); 2 Laboratory for Chemistry and Surface Modification, Hochschule Osnabrück University of Applied Sciences, Albrechtstraße 30, 49076 Osnabrück, Germany; s.petersen@hs-osnabrueck.de

**Keywords:** abrasion, dentin, brushing, charcoal, tooth wear, toothbrush, toothpaste

## Abstract

The aim of this study was to investigate abrasion on human dentin after brushing with activated charcoal toothpastes. A self-designed brushing machine was used to brush five groups (Group A: Water, Group B: Sensodyne Pro Schmelz, Group C: Splat Blackwood, Group D: Curaprox Black is White, and Group E: Prokudent Black Brilliant) with electrically powered toothbrushes for 4 h. The abrasive dentin wear was calculated using profilometry data. Furthermore, thermogravimetric analyses and scanning electron microscopy were used to analyze the composition of the toothpastes. Mean dentin loss by brushing were (71 ± 28) µm (Splat Blackwood), (44 ± 16) µm (Curaprox Black is White), (38 ± 13) µm (Prokudent Black Brilliant), (28 ± 14) µm (Sensodyne Pro Schmelz), and (28 ± 13) µm (Water). Groups A/B/D/E and group C each lie in one subset, which is statistically different from the other subset according to a post hoc Tukey test (*p* = 0.05). Within the limitations, it can be concluded that the content of activated charcoal in charcoal toothpastes had little influence on the observed abrasive behavior, although one of the charcoal toothpastes showed the highest abrasion on dentin.

## 1. Introduction

Oral and dental health is a necessary component of a person’s overall health and well-being. It includes sound teeth and supporting tissues. A healthy oral cavity can help maintain a healthy body, while negative effects on the whole organism may result if maintenance is poor [1]. Various dental care products are commercially available for the maintenance of oral hygiene. There are various manual and electric toothbrushes, as well as different toothpastes. Marketing of these hygiene products includes public personalities (as from social media) and setting trends with new products. Among the current trends are toothpastes containing activated charcoal. The manufacturers of such toothpastes advertise a whitening effect that is supposed to make teeth appear whiter after repeated use, due to the removal of extrinsic stains. Greenwall et al. [2] reported that activated charcoal has been suggested to bind to all tooth surface deposits, which were then brushed away and supposedly left tooth surfaces free of any deposits. The authors were unaware of any supporting data for this claim. Aside from this binding mechanism, increased abrasion might play a role for stain removal, which can unfortunately also imply unwanted abrasion of tooth substances.

Some published studies are critical of these toothpastes [2,3,4], for reasons including the lack of fluoride content [2,3], the unproven whitening effect [4,5,6], and the possibly high abrasion behavior [4,5]. The authors suggested possible health risks [2,3], such as the potential increased risk of caries when using non-fluoridated toothpastes [3]. There is evidence that toothbrushing with whitening toothpastes can cause gingival injury and recession [7], and abrasion of tooth structure [8,9].

Mechanical and/or inflammatory influences can lead to gingival recession, so that dentin is exposed [7]. Thus, dentin and enamel may be affected during tooth brushing. Due to its anatomy and physiology, dentin is shaped differently to enamel. Enamel is more mineralized and harder than dentin [10]. Several methods have been published to determine the abrasion of dentin. Some are limited only to the behavior of abrasion without pre-treatment of dentin [11,12], others to abrasion and the additional erosion of dentin [13,14,15]. Pertiwi et al. found that charcoal-containing toothpaste can increase surface roughness [16]. 

In this study, the abrasion behavior of four different toothpastes on human dentin was investigated in vitro, using a brushing machine with electric toothbrushes that was described elsewhere [17]. Abrasive dentin wear was computed from tactile profilometry data. Furthermore, the solids content of the toothpastes was analyzed by thermogravimetric analysis and electron microscopy. 

The underlying research questions of this study are: Do activated charcoal toothpastes have a significantly higher dentin abrasiveness than a conventional toothpaste with low abrasiveness? If yes, is this caused by the charcoal content?

## 2. Materials and Methods

Human dentin was brushed with different toothpastes as in our previous study, but with a focus on enamel abrasion [17]. Deionized water was used in group A. A toothpaste without charcoal was used in group B (*Sensodyne ProSchmelz,* GlaxoSmithKline Healthcare Consumer, Brentford, UK). Additionally, three activated charcoal toothpastes were examined: In group C, *Curaprox Black is White* (Curaden, Kriens, Switzerland); in group D, *Blackwood* (Splat, Moskau, Russia); and in group E, *Prokudent Black Brilliant* (Rossmann, Burgwedel, Germany). The conventional toothpaste (Group B) is a low abrasiveness toothpaste, thus expected a priori to have a comparable low silica content and/or particle size and shape that is beneficial. The factors that were expected to have the most impact on abrasiveness for Groups C, D, and E were charcoal and silica content, both of which were unknown a priori. A full list of substances, including the clinically relevant fluoride content, is given in Table 1. The batch numbers and expiration dates were not provided consistently on toothpaste packaging; in order to avoid possible misinterpretation, we provide the data, as printed. Furthermore, toothpastes for Groups B, C, and E have a symbol that indicates that the toothpastes should be used within 12 months, while the toothpaste for group D indicated 6 months.

### 2.1. Sample Preparation

Caries free human molars were gathered anonymously at an external dental office and later used to prepare 45 samples. After cleaning, the teeth were stored in Chlorhexidine (Dynexidin Forte 0.2%, Keussler Pharma, Wiesbaden, Germany). After storing the teeth for 7 days in deionized water at 37 °C ± 1 K, teeth were cut in the mesiodistal and buccolingual directions. For cutting, a cylindrical diamond grinder (HS-Maxima^®^ Diamant Flame, Form 863, Henry Schein Inc., Melville, NY, USA) was used under water cooling. To expose the dentinal surface, the enamel was ground down with the grinder until the dentin–enamel junction was exposed, which was controlled by light microscopy. The dentin was exposed on the buccal or lingual side, and 45 molar dentin pieces were produced and embedded in PMMA (PalaPress, Kulzer, Hanau, Germany). The whole dentin piece was covered in order to generate a flat disc. Using a polishing disc (Silicon carbide disc of 45 µm, Buehler, Esslingen am Necker, Germany), the dentinal side of this disc was polished until a small area of dentin was exposed. A part of this initially exposed area was later used for the measurement of substance loss. Small marks for subsequent orientation were cut into the margin of the disc with a scalpel, followed by brief polishing of the dentin sample with silicon carbide (grain 2400, Struers, Copenhagen, Denmark). As described previously [17], all samples were randomly assigned to five groups. The number of samples per group (*n* = 9) was chosen according to the previous experiments for enamel abrasion [17], as this was seen as a workable compromise between statistical power and available time resources. The effect size of different toothpastes for brushing on dentin was expected to be higher due to more (absolute) abrasive material wear, but the uncertainty caused by the measurement method was smaller (relative to the material wear). The standard deviation for dentin abrasion was expected to be higher due to the inhomogeneous structure of dentin, but unknown for our test setup.

### 2.2. Profilometry and Calculation of Substance Loss

The implementation of profilometry and calculation of substance loss was given elsewhere [17] and performed in the same way in this study. In short, the abrasive dentin wear was calculated via a Matlab script as the mean height difference between a scan before brushing and a scan after brushing.

### 2.3. Brushing

The specimens of each group (*n* = 9) were attached to a self-built brushing machine, also described previously [17], with the dentin surface positioned upwards. Four hours of brushing by electrical rotating brushes (Oral-B CrossAction, Procter&Gamble, Schwalbach am Taunus, Germany) were performed under a load of 150 g to simulate 4 years of brushing [18]. All specimens were immersed in a slurry prepared with toothpastes and deionized water, in a 1:2 weight ratio [19]. In each brushing chamber, the slurry was pumped in and out using a peristaltic pump (IPC Ismatec, Cole-Parmer, Wertheim, Germany). After brushing, the masking tape was removed, and the specimens cleaned of any residue with ethanol (70%), before thorough rinsing with deionized water.

### 2.4. Thermogravimetric Analysis and REM Analysis

A thermogravimetric analysis (TGA) was carried out to examine the abrasive particles of the toothpastes. The toothpaste slurry was diluted with deionized water (1:3 weight ratio) and centrifuged at 450 rpm for 20 min (Thermo Multifuge 1S-R, Heraeus, Hanau, Germany). Following this, the solids were dried in a warming cabinet (Universal warming cabinet, Loading Modell 100–800, Memmert, Schwabach, Germany) for 72 h. Each of the dried solids were heated up to 900 °C in porcelain crucibles in a thermogravimetric furnace (TGA/SDTA 821e, Mettler-Toledo, Gießen, Germany). Between the temperatures from 25 °C to 650 °C, a nitrogen atmosphere was used, but above 650 °C, an oxygen atmosphere was used in order to burn the carbon content. The solid residue from the crucible was pulverized by a glass spatula and placed on aluminum stubs covered with self-adhesive film. The samples were sputter-coated with gold (Q150RES, Quorum Technologies Ltd., Laughton, UK, 20 mA, 300 s). One sample of each toothpaste was analyzed by scanning electron microscopy (Crossbeam 540 Gemini 2, Carl Zeiss, Oberkochen, Germany, 1000× and 10,000×, 10 kV), and an EDX microanalysis (20 kV, 1000×, Version 4.4, Ametek, Weiterstadt, Germany) was performed to determine the presence of chemical elements in the dentifrices. To obtain a rough estimate of the particle size we superimposed the REM images onto a grid with sizes of 10 µm, 20 µm, or 30 µm, in order to check whether the particles fit inside a grid with the given sizes. TGA as well as REM analysis were each performed with one sample, so the analysis has a qualitative character.

### 2.5. Statistics

A univariate ANOVA including a Tukey HSD post hoc test was performed using Minitab (Minitab, München, Germany) at a significance level of 0.05 to check the results for statistical differences.

## 3. Results

**Figure 1:**
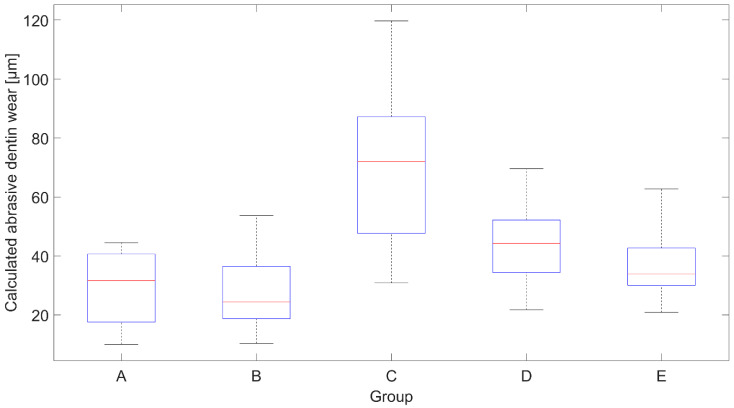
Calculated abrasive dentin wear after brushing. The red line indicates the median wear. The bottom and top edges of the box indicate the 25th and 75th percentiles, respectively, and the whiskers extend to the most extreme data points. Groups are A: deionized water, B: Sensodyne Pro Schmelz, C: Splat Blackwood, D: Curaprox Black is White, and E: Prokudent Black Brilliant.

Figure 1 shows the calculated abrasive dentin wear. According to a post hoc Tukey HSD test (*p* = 0.05), the results for Group A/B/D/E and Group C each lie in a subset that differed statistically significantly from the other subset. That means that only the pairwise comparisons with Group C were statistically different. For a more convenient comparison, the RDA value, mean abrasive dentin wear, and standard deviation (SD) are given in Table 2 for enamel and dentin. 

**Figure 2:**
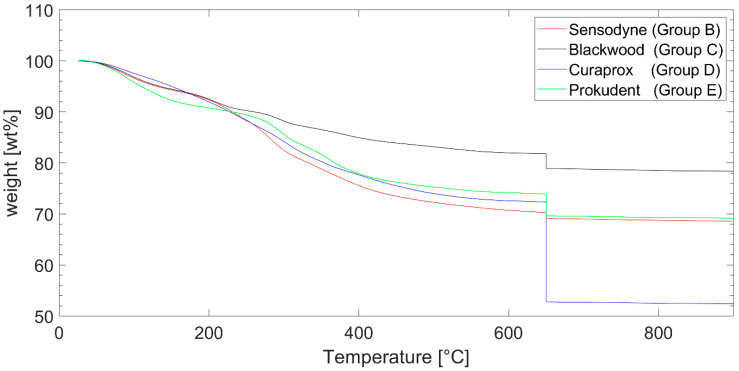
Weight loss during thermogravimetric analysis (TGA) of the groups. Nitrogen atmosphere was used from 25 °C to 650 °C. Above 650 °C, the chamber was flooded with oxygen.

The thermogram in Figure 2 shows the weight loss of the solid residues generated by centrifugation of the toothpastes dependent on the chamber temperature. From 25 °C until 650 °C, a nitrogen atmosphere was used. In this atmosphere, organic components are pyrolyzed or evaporate. Starting at 650 °C, the atmosphere was switched to oxygen. When burning in oxygen started, a sharp drop in weight was noticeable due to burning of pyrolytic carbon and activated charcoal. The graphs of the toothpastes with charcoal run steeply downwards above 650 °C under the oxygen atmosphere. In Table 3, the calculated amount of charcoal and inorganic substances are given. For charcoal toothpastes, the weight loss above 650 °C was attributed to burning of activated charcoal, as it could not be distinguished from burning pyrolyzed carbon. 

To determine the composition, one sample of each toothpaste residue after TGA was analyzed via EDX. In all dentifrices, silicon (Si) and oxygen (O) were dominant. Other elements above 1 wt% were: 2.2 wt% potassium and 1 wt% titanium for Sensodyne (Group B); 1.1 wt% calcium for Curaprox (Group D); and 2.5 wt% sodium, 1.5 wt% phosphorus and 1.4 wt% potassium for Prokudent (Group E). The residue consists mostly of silica particles, which act as an abrasive material.

**Figure 3:**
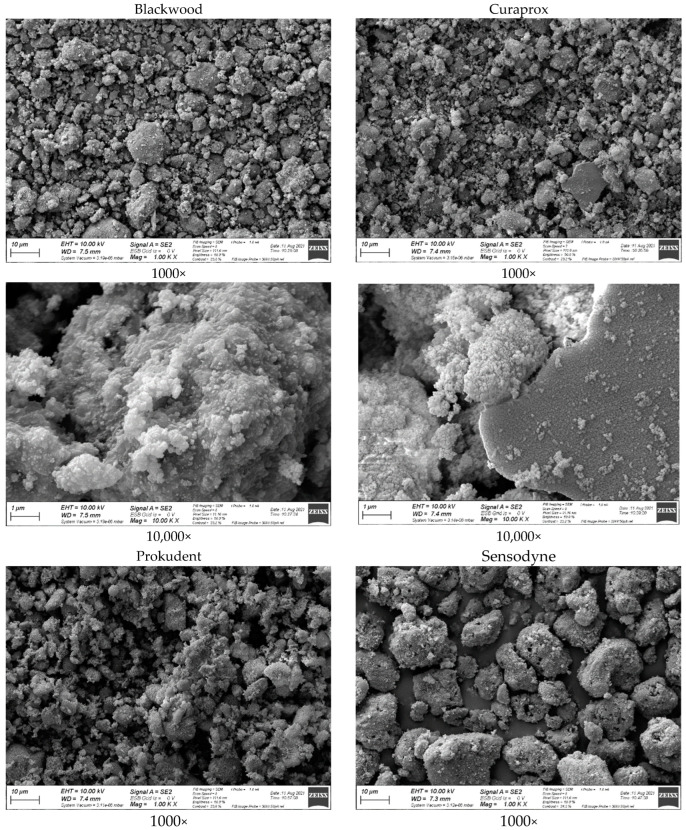

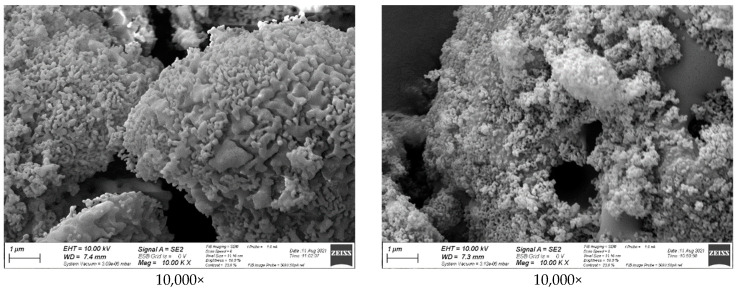
SEM micrographs of inorganic residues (silica) after TGA.

The SEM images of inorganic residues (mostly silica) after the TGA are presented in Figure 3. The silica particles differed in size, surface morphology, and shape. For Sensodyne (Group B), 21 particles that fit in a 20 µm grid (and are >10 µm) and 2 particles that fit in a 30 µm grid were identified. In comparison to the other toothpastes, there only seemed to be a relatively small number of particles <10 µm. For Blackwood (Group C), six particles fitting in a 20 µm grid were identified; in addition, there were many particles fitting in the 10 µm grid. For Curaprox (Group D), the observations were similar. There were also many particles <10 µm, and four particles fitting only in the 20 µm grid, whereas one of these particles was noticeably sharper in appearance. For Prokudent (Group E), a different sponge-like surface structure of the particles was observed. There were six particles fitting only in the 20 µm grid, and many particles <10 µm. The particles seem somewhat bigger on average than for Groups C and D.

## 4. Discussion

The results show that one of the activated charcoal toothpastes led to higher abrasion on dentin than the conventional toothpaste and water, which is consistent with the abrasion behavior on enamel that was observed by Greuling et al. [17]. The highest mean abrasive dentin wear was 71 µm ± 25 µm in 4 h of electric brushing. On the assumption of 10 s brushing/day on a single area and without further influences (nutrition, remineralization, etc.), this leads to 1400 µm dentin loss in 80 years. This is, of course, only a rough theoretical estimation. The clinical situation will differ because of different boundary conditions.

Overall, the abrasive dentin wear of the toothpaste with the highest wear was about 15 times higher than on enamel using the same paste and approach [17]. It is widely known that brushing on dentin leads to higher abrasion than brushing on enamel [24]. Dentin is composed of micrometer-sized tubules, which are surrounded by highly mineralized peritubular dentin that is embedded within a partially mineralized collagen matrix, the intertubular dentin [25]. These tubules run from the enamel–dentin junction to the pulp, whereas the tubule density is greater closer to the pulp than at the enamel–dentin interface [26]. This dentin microstructure has consequences for the hardness. Kinney et al. found that peritubular dentin was significantly less hard than enamel, but several times harder than intertubular dentin [27]. The dentin close to the enamel is also called mantle dentin and can be distinguished from circumpulpal dentin by staining [28]. In our approach, using light microscopy without staining, we were able to distinguish between enamel and dentin, but not between different dentin types. Consequently, we have brushed dentin samples that may have differed considerably in hardness, even for samples from the same patient, which may explain why the observed standard deviations were considerably higher than for enamel.

In regards to the statistical results, one might wonder why only Group C appears statistically different. Is there really no difference between brushing with water and lower abrasive toothpaste? The answer is that our data cannot demonstrate such a difference. It does not mean that there is no difference, but that the difference is so small, that it can not be shown given the rather high standard deviation in the data. As noted before, the main reason for the high standard deviation can perhaps be due to differences in enamel hardness, preparation depth, and orientation of the dentin tubules and between individual patients. One might further ask why does brushing with water show abrasion at all? Is it just the brush that leads to damage? It might be that removed dentin particles had not moved away from the brushing side and played a role in the abrasive interplay between the sample and brush. However, this is speculation and not supported by the data. Further work is needed to clarify this. A look at the time behavior of dentin abrasive wear might help. For instance, is the abrasive wear proportional to the brushing time? The data in Table 2 suggests that high relative dental abrasion (RDA) correlates with high enamel abrasion. However, this only applies to Groups B, C, and D, and the RDA data for Group E did not fit that trend. Lack of agreement between profilometry data and radiotracer data was already reported in the literature [29].

With respect to the abrasive properties of the toothpaste, it is of interest if the activated charcoal content has a noticeable influence on the observed abrasion. Therefore, self-mixed slurries according to ISO 11609 were used with different contents of activated charcoal and silica in preliminary experiments (not presented here). Unfortunately, the preliminary experiments gave significant sedimentation problems in the brushing machine, which could not be fully solved in the available study time for technical reasons. The toothpaste–water slurries presented in this study do not show these problems. While one might expect the solid contents to play the main role in abrasion, substances that alter the manner in which the solid particles stick to the brush or are distributed (e.g., foam building substances) might also have a significant influence on abrasion. However, we cannot provide more details about that influence based on the available data.

Besides the abrasive properties of the toothpaste, the choice of the toothbrush influences the abrasive wear as well as the cleaning properties. Welss et al. [30] compared abrasive wear on enamel and dentin for a manual and electric toothbrush using the same brushing duration, same brush tension, and same source of specimens. When brushing with an electric brush, they found a 3- to 5-fold increase in wear on enamel and a 3- to 6-fold increase in wear on dentin. However, for clinical comparison, one should keep in mind that patients do not necessarily handle manual and electric brushes in the same way, especially as some electric brushes have force sensors and warn the user when excessive force is applied. Furthermore, different types of brushing heads might also lead to different results for wear. Thus, this kind of comparison is strongly dependent on the scenario.

Figure 3 compares the size and shape of the silica particles. There seems to be no unique feature of Blackwood (Group C) which could explain the observed higher abrasion. Compared to Groups B and E, Groups C and D show relatively small particles. For two-body and three-body abrasion, it is well known that greater particle size led to higher abrasion for blunt particles with particle size below 100 µm [31]. Therefore, particle size alone cannot explain why Group C shows higher abrasion. Braig et al. recently discussed the possibility that particle agglomeration on the toothbrush filament can also play a role, which can be controlled via the use of dispersants [32]. It is possible that this particle agglomeration also played a role here.

The results from the TGA analysis (and EDX) show inorganic residue that are mostly silica, between 15 wt% and 28 wt%. The typical amount of silica as abrasive agent in toothpastes has been reported to be in the range of 8 wt% to 20 wt% [33]. The toothpaste without activated charcoal shows only a small decline in the TGA burning phase, which can be expected to be due to pyrolyzed organic compounds. The toothpaste with the highest abrasion in the current study (Group C) also showed the highest amount of silica. On the other hand, there was toothpaste (Group D) that contained ≈5 times as much activated charcoal as Group C but showed lower abrasion. Thus, it does not seem that the abrasive behavior was dominated by content of activated charcoal. The data suggested that silica played a more important, or even dominant, role. However, to support this conclusion, it would be beneficial to test different concentrations of activated carbon and silica in the future. 

Moreover, further research is needed to clarify the whitening effect and plaque removal of these activated charcoal toothpastes.

## 5. Conclusions

Within the limitations of this study, it was concluded that the content of activated charcoal in charcoal toothpastes had little influence on the observed abrasive behavior, although one of the charcoal toothpastes showed the highest abrasion on dentin. As expected, brushing on dentin showed much higher abrasion than on enamel. Thus, it is generally advisable to use low-abrasion toothpastes when brushing on exposed dentin cannot be avoided. 

## Figures and Tables

**Table 1:** Information about the ingredients of the toothpastes used. The ingredients are the same as in our previous work [17].

Group	Commercial Brand	Ingredients ^1^	Expiration Date/Batch Number as Given on Toothpaste Packaging
A	-	Aqua	-
B	Sensodyne Pro Schmelz Repair Zahnschmelz, conventional (no charcoal)	Hydrated Silica, Aqua, Sorbitol, Glycerin, Potassium Nitrate, PEG-6, Sodium Lactate, Cocamidopropyl Betaine, Aroma, Titanium Dioxide, Xanthan Gum, Sodium Saccharin, Sodium Fluoride (1450 ppm F^−^), PVM/MA Copolymer, Sodium Hydroxide, Limonene	EXP 200823 02942KWA
C	Splat Blackwood	Hydrated Silica, Charcoal Powder, Aqua, Hydrogenated Starch Hydrolysate, Glycerin, Maltooligosyl Glucoside, Sodium Lauroyl Sarcosinate, Cellulose Gum, Aroma, Capryloyl/Caproyl Methyl Glucamide, Lauroyl/Myristoyl Methyl Glucamide, Sodium Benzoate, Stevia Rebaudiana Leaf Extract, Potassium Sorbate, Menthol o-Cymen-5-ol, Juniperus Communis Sprout Extract, Limonene	R02 EXP 12.23
D	Curaprox Black is White	Hydrated Silica, Charcoal Powder, Aqua, Sorbitol, Glycerin, Aroma, Decyl Glucoside, Cocamidropropyl Betaine, Sodium Monofluorophosphate 950 ppm F^−^, Tocopherol, Xanthan Gum, Maltodextrin, Mica, Hydroxylapatite (Nano), Potassium Acesulfame, Titanium Dioxide, Microcrystalline Cellulose, Sodium Chloride, Citrus Limon Peel Oil, Sodium Hydroxide, Zea Mays Starch, Amyloglucosidase, Glucose Oxidase, Urtica Dioca Leaf Extract, Potassium Thiocyanate, Cetearyl Alcohol, Hydrogenated Lecithin, Menthyl Lactate, Methyl Diisopropyl Propionamide, Ethyl Menthane Carboxamide, Stearic Acid, Mannitol, Sodium Bisulfite, Tin Oxide, Lactoperoxidase, Limonene	400MHDEXP1021
E	Prokudent Black Brilliant	Hydrated Silica, Charcoal Powder, Aqua, Sorbitol, Propylene Glycol, Pentasodium Triposphate, Tetrapotassium Pyrophosphate, Sodium C14-16 Olefin Sulfonate, Aroma, Disodium Pyrophosphate, Xanthan Gum, Menthol, Sodium Fluoride (1450 ppm F^−^), Sodium Saccharin	025377 B

^1^ Information according to the manufacturer’s label.

**Table 2:** RDA, mean abrasive wear and standard deviation (SD) for dentin and enamel (taken from previous work [17]).

Group	RDA	Mean Abrasive Dentin Wear [µm]	SD Abrasive Dentin Wear [µm]	Mean Abrasive Enamel Wear [µm]	SD Abrasive Enamel Wear [µm]
A	-	28 ^a^	13	1.71	0.63
B	35 ± 15% [20]	28 ^a^	14	2.32	0.69
C	75 [21]	71 ^b^	28	4.55	0.60
D	50 [22]	44 ^a^	16	3.25	0.85
E	120 [23]	38 ^a^	13	1.45	0.61

Groups are A: deionized water, B: Sensodyne Pro Schmelz, C: Splat Blackwood, D: Curaprox Black is White, and E: Prokudent Black Brilliant. Different group indices indicate a statistically significant difference.

**Table 3:** Calculations of the activated charcoal and inorganic content according to the TGA measurements. The ‘total solids content’ refers to the solid content after centrifugation and drying divided by the weight of the used toothpaste. ‘Weight loss above 650 °C’ and ‘Inorganic residue after TGA’ refer to the ‘total solids content’. The last two columns are given relative to the toothpaste weight.

Group	Toothpaste Total Solids Content [wt%]	Weight Loss above 650 °C [wt%]	Inorganic Residue after TGA [wt%]	Activated Charcoal Content in Toothpaste [wt%]	Inorganic Content in Toothpaste [wt%]
B	27.62	1.20	68.59	-	18.95
C	36.60	3.35	78.37	1.23	28.68
D	30.21	19.75	52.40	5.97	15.83
E	21.81	4.50	69.18	0.98	15.08

Groups are B: Sensodyne Pro Schmelz, C: Splat Blackwood, D: Curaprox Black is White, and E: Prokudent Black Brilliant.

## Data Availability

The data are available on reasonable request.
